# Recent advances in deep learning and language models for studying the microbiome

**DOI:** 10.3389/fgene.2024.1494474

**Published:** 2025-01-07

**Authors:** Binghao Yan, Yunbi Nam, Lingyao Li, Rebecca A. Deek, Hongzhe Li, Siyuan Ma

**Affiliations:** ^1^ Department of Biostatistics, Epidemiology, and Informatics, Perelman School of Medicine, University of Pennsylvania, Philadelphia, PA, United States; ^2^ Department of Biostatistics, Vanderbilt University Medical Center, Nashville, TN, United States; ^3^ School of Information, University of South Florida, Tampa, FL, United States; ^4^ Department of Biostatistics and Health Data Science, University of Pittsburgh, Pittsburgh, PA, United States

**Keywords:** microbiome, virome, artificial intelligence, large language models, transformer, attention

## Abstract

Recent advancements in deep learning, particularly large language models (LLMs), made a significant impact on how researchers study microbiome and metagenomics data. Microbial protein and genomic sequences, like natural languages, form a *language of life*, enabling the adoption of LLMs to extract useful insights from complex microbial ecologies. In this paper, we review applications of deep learning and language models in analyzing microbiome and metagenomics data. We focus on problem formulations, necessary datasets, and the integration of language modeling techniques. We provide an extensive overview of protein/genomic language modeling and their contributions to microbiome studies. We also discuss applications such as novel viromics language modeling, biosynthetic gene cluster prediction, and knowledge integration for metagenomics studies.

## 1 Introduction

The study of microbiomes and metagenomics has significantly advanced our understanding of microbial communities and their complex interactions within hosts and environments. The microbiome refers to the collective genomes of microorganisms residing in a specific habitat, such as human body sites (e.g., gut, skin, airway) and environments (e.g., air, soil, water). Metagenomics research involves the direct profiling and analysis of these microbial communities’ genomic sequences, bypassing the need for isolating and culturing individual members. This approach allows for a comprehensive assessment of microbial diversity, functions, and dynamics within their natural contexts.

The complex dependency encoded in metagenomic sequences represents gene/protein-, organism-, and community-level biological structures and functions. Examples include residue-residue contact patterns for protein 3D structures, functional relationship between genes and their regulatory, non-coding counterparts (e.g., promoters, enhancers), mobility for horizontal gene transfers, and genome-scale organization of functional modules (e.g., operons and biosynthetic gene clusters). Such dependency patterns, when interrogated at the revolutionary scale (i.e., encompassing many diverse organisms and environments), can capture fundamental biological properties as shaped over time by evolutionary processes, thus representing a meaningful “language of life”. On the other hand, the availability of microbial genomic sequences, both annotated and unannotated for biological properties [e.g., UniRef, Suzek et al. (2007); MGnify; Richardson et al. (2023)], drastically increased over the past decade due to advancements in next-generation sequencing protocols, bioinformatics, and computational capacities. The availability of these metagenomic “big data” suggests that, given capable modeling architecture and capacity, microbiomes’ evolutionary and functional dependency structures can be computationally learned, represented, and utilized for studying the microbiome.

**FIGURE 1:**
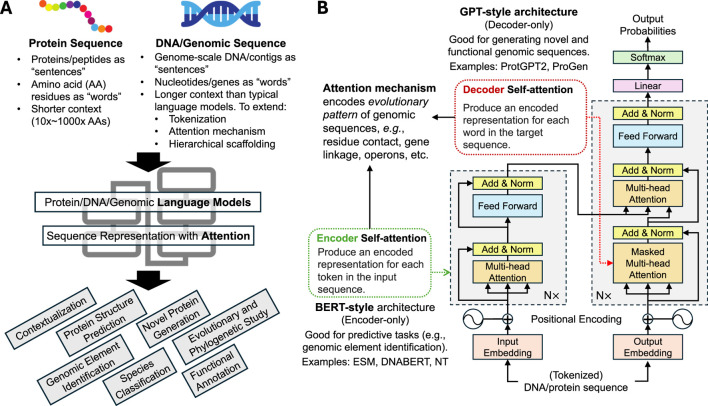
Review of protein/DNA/genomic language models as applied to metagenomic studies. **(A)** Protein and genomic sequences share similar properties as natural language sequences, with amino acids or neucleotides as units of sequences (“tokens”). The complex dependency structure of protein/gene-level or genomic-scale sequences can then be modeled by language model techniques, such as transformer-based attention mechanism for various downstream tasks. **(B)** Review of encoder- and decoder-style transformer attention mechanisms and their applications in metagenomic studies. Decoder-style model architecture (similar to that of BERT) aims to provide a meaningful representation of genomic sequences and is useful for downstream predictive tasks. Encoder-style model architecture (similar to that of ChatGPT) generates new sequences given past tokens and is most useful for generative tasks such as novel protein design.

To this end, advances in powerful artificial intelligence (AI) methods regarding the design and training of highly complex, large-scale deep learning models have been adopted to characterize microbial genes and genomes from large-scale metagenomic sequences, offering powerful tools for extracting, interpreting, and integrating complex microbiome data (Hernández Medina et al., 2022). In particular, inspired by the recent breakthrough of large language models (LLMs) in dealing with natural language tasks, similar methods have been developed and applied for modeling protein and genomic languages of life. To avoid conflating nomenclature, we reserve “LLM” in this review exclusively for large language models [e.g., ChatGPT (Liu Y. et al., 2023)] and instead use terms such as “protein language model” and “DNA language model” to more explicitly refer to genomic sequence models. Indeed, whereby natural languages are organized in sequential words and phrases which form the basic units of modeling (“tokens”), microbial genomic elements are similarly organized as sequences of nucleotide base pairs (for genomic DNA) or amino acids (AA, for proteins). Given the complexity of genomic dependency structures, metagenomic research was fast to adopt advanced language modeling techniques for studying microbial community sequences, with models spanning different genomic scales (microbial proteins, contigs, genomes, and communities) and designed for a variety of tasks (Figure 1), yielding promising performance improvement and novel applications.

This review aims to provide a survey of recent developments in deep learning and language modeling for analyzing microbiome and metagenomics data. We focus on problem formulation, the datasets required to address these questions, and how sequence-based language models are integrated into deep learning algorithms. In Section 2, we briefly discuss the typical language model architecture from recent LMM breakthroughs and how they can be applied towards genomic sequence modeling. We discuss in Section 3 two broad classes of language models for microbiome studies, namely, *protein* language models and *DNA/genomic* language models, distinguished by their drastically different range of genomic “contexts” for sequence dependency structures. We then review three specific applications of high interests to the field in Sections 4–6, namely, novel viromics language modeling, models for predicting biosynthetic gene clusters (BGCs), and knowledge integration in metagnomics studies aided by natural language LLMs. We conclude with prospective remarks and discussions in Section 7.

## 2 Brief review of LLMs and their extension towards modeling the language of life

LLMs are advanced foundation models specifically designed to understand and generate human language. They can perform a wide range of natural language processing (NLP) tasks, such as question-answering, information extraction, and text summarization (Chang et al., 2024; Li et al., 2024). The scalability, versatility, and contextual understanding of LLMs can be attributed to two key factors. First, LLMs are trained on massive datasets that encompass diverse linguistic patterns, enabling them to learn complexities and nuances in languages as sequences of tokens (i.e., words and phrases). Second, LLMs are built on the transformer architecture, which consists of an encoder and a decoder and uses self-attention mechanisms to process input sequences. The attention mechanism efficiently encodes dependency structures of sequential tokens, vastly increases the learnable lengths of long-range dependencies, and encodes tokens and sequences accounting for their upstream and downstream neighboring “contexts”. This allows for efficient processing of sequential data, enabling LLMs to provide meaningful representation of input text and generate coherent and contextually relevant output text based on input prompts (Vaswani et al., 2017).

Inspired by LLMs, language models in microbiome research often employ a similar architectural design (Figure 1). These language models of genomic sequences (Ligeti et al., 2024; Shang et al., 2023; Mardikoraem et al., 2023) thus provide an improved representation of sequences with richer context and can be scaled up to and trained at impressive complexities (up to billions of model parameters). Such models often include a transformer encoder component that processes input sequences—such as protein or DNA sequences—and converts them into high-dimensional representations that capture essential features of input sequences in their contexts. The attention mechanism in these models assigns different weights to various parts of the sequence, characterizing dependency structures shaped over evolution and allowing the model to focus on relevant regions. This focus ensures that the model prioritizes areas within a sequence that are most significant for biological interpretation. Similar to the application of BERT for various natural language tasks (Devlin, 2018), the encoded representation can then be used for tasks such as contextualizing microbial genes and genomic sequences in their broader genomic neighborhoods (Hwang et al., 2024), predicting the structure and functions of protein given their sequences (Lin et al., 2023), and segmentation and identification of specific regulatory elements across microbial genomes (Zhou et al., 2023). In comparison, decoder-style models focus on generating output sequences, given the encoded representation of past sequence tokens. This is more similar to GPT-style LLMs (Brown, 2020), whereby the task towards microbiome application often involves the generation of new, functional, and viable protein sequences (Ferruz et al., 2022; Madani et al., 2023; Jin et al., 2024).

## 3 Language modeling of proteins, contigs, and genomes of the microbiome

To facilitate the survey, we categorize existing language models for metagenomic sequences into two classes: (1) models on the protein/gene scale and (2) those on the genome scale. The first, which we term *protein* language models (Table 1), fits well within the context length for transformers since microbial proteins are generally under 1,000 AAs (tokens). In contrast, *DNA* or *genomic* language models (Table 2) often require additional techniques to extend their operating ranges due to the large scale of microbial contigs or whole genomes. For example, the bacterial genome typically ranges from 0.5 to 10 million base pairs, a scale that often far exceeds the context window of transformers. In addition, the two classes target different applications: protein language models are used for designing and predicting individual proteins, while DNA/genomic language models examine genes and proteins within their broader genomic contexts as well as intergenic regions.

**TABLE 1:** Protein language models.

Model	Model architecture	Usage	Relevance to the microbiome	Additional notes
Antimicrobial peptide (AMP) prediction Ma et al. (2022)	The best combination of LSTM, attention-based and BERT models	Identifying candidate AMPs from human microbiome data that were further validated experimentally	Training/validation data and application focus on bacterial peptides	AMPs predicted through best ensemble of different prediction models, suggesting robustness of findings
ProtGPT2 Ferruz et al. (2022)	Transformer decoder model that matches that of GPT2	Generating novel proteins	Universal training and validation sequences include microbial proteins	Byte Pair Encoding (BPE) tokenization improves model performance
ProGen Madani et al. (2023); Progen2 (Nijkamp et al., 2023)	Standard transformer decoder with left-to-right causal masking	Generating viable and novel proteins with controlled functions	Universal training and validation sequences includes microbial proteins Generated viable and experimentally validated antibacterial proteins	Model size study suggests even huge models ( > 6 billion parameters) are far from overfitting. Suggested model can traverse protein space underexplored in naturally observed sequences
ESM-1b Rives et al. (2021); ESM-2, ESMFold (Lin et al., 2023)	ESM-1b/ESM-2: BERT-like masked token architecture; ESMFold: Folding NN (based on ESM-2 representations) composed of folding blocks + structure prediction module	ESM-1b/ESM-2 provides meaningful sequence representations ESMFold provides fast and accurate prediction of protein 3D structure based on sequences	Universal training and validation sequences include microbial proteins Provides database with prediction of large number of metagenomic protein sequences	Protein sequence attention pattern can predict structural residue contact probability in a zero-shot fashion Language model architecture allows faster structure prediction compared to SOTA models based on multiple sequence alignment

**TABLE 2:** DNA/genomic language models.

Model	Model architecture	Usage	Relevance to the microbiome	Additional notes
DNABERT Ji et al. (2021); DNABERT-2 (Zhou et al., 2023)	Encoder-only transformer architecture with masked modeling DNABERT-2 incorporates new techniques such as Attention with Linear Biases to increase context length and Flash Attention to increase computation and memory efficiency	Human (DNABERT) and multi-species (DNABERT-2) for representing genomic nucleotide sequences applicable for downstream tasks Demonstrated utilities in various tasks: genomic element prediction (promoter, enhancer, transcription factor, epigenetic marks), microbial species classification	DNABERT-2 training/validation based on multi-species genomes including bacteria and fungi Evaluated for microbial gnomic element prediction and species classification	Also provides system of benchmarking tasks for evaluating DNA language models BPE tokenization improves model performance
gLM Hwang et al. (2024)	RoBERTa-based transformer architecture Genes (embedding from ESM-2) are tokens and microbial contigs (15–30 genes) are sequences	Enriching microbial genes’ representations with longer-range genomic context. Providing contextualized gene function prediction and characterizing higher-order genomic features	Training/validation data and application focus on microbial genomes	“Contextualization” learns representations of microbial genes in their longer-range genomic contexts, encoding enriched genomic information such as mobility for horizontal gene transfer and operon membership
NT Dalla-Torre et al. (2024); SegmentNT (de Almeida et al., 2024)	NT: encoder-only transformer architecture with masked modeling SegmentNT: a segmentation NN head based on NT embedding	NT provides human-focused and multi-species (DNABERT-2) representation for genomic nucleotide sequences applicable for downstream tasks SegmentNT specializes in predicting genomic elements based NT representation	NT has multi-species version incorporating bacterial and fungal genomes SegmentNT does not incorporate microbial genomes	Performance of multi-species model was demonstrated to match or outperform human-only model on tasks specific for human genomes
Species-aware DNA language models Karollus et al. (2024)	Standard DNABERT architecture was adopted	(a) to learn meaningful species-specific and shared regulatory features across evolution (b) to transfer these features to unseen species	Trained on non-coding regions from > 800 fungal species spanning over 500 million years of evolution	Focus on non-coding DNA and regulatory elements
FGBERT Duan et al. (2024)	Joint objectives of (a) masked gene modeling with a context-aware tokenizer and (b) contrastive learning with data augmentation and negative sampling to capture the functional relationships between genes	Downstream tasks include gene operons, functional genes, genome pathogenes, and nitrogen cycle prediction	Pre-trained on 100 million metagenomic sequences	First metagenomic pre-trained model encoding (a) context-aware and (b) function-relevant representations of metagenomic sequences. Protein-based gene representations converted from the DNA sequence from metagenomic sequences, to protein sequence using ENA, and then to ESM-2 representations
ProkBERT family Ligeti et al. (2024)	Encoder-only masked language modeling with the newly introduced Local Context-Aware (LCA) tokenization	Generate nucleotide sequence representation. Applied downstream tasks include (a) bacterial promoter prediction and (b) bacteriophage identification	Bacteriophages have a significant role in the microbiome, influencing host dynamics and serving as essential agents for horizontal gene transfer	The implementation of masked language modeling (MLM) with LCA requires slight variations in masking tokens: to prevent trivial restoration from locality, the model needs to ensure neighboring tokens to be masked as well

### 3.1 Protein language models for novel protein generation

Existing protein language models applied towards microbiome studies are summarized in Table 1. We highlight two specific applications, namely, the generation of novel proteins and the prediction of their functions and structures. The dependency structure of amino acids across known microbial proteins is learned and utilized to generate artificial, potentially novel protein sequences by protein language models such as ProGen (Madani et al., 2023) and ProtGPT2 (Ferruz et al., 2022). This is performed in an autoregressive fashion, often with decoder-only architecture similar to that of the GPT language models, whereby the likely AA at the next position is predicted given the sequence of preceding residues. If trained across a sufficiently large variation of raw occurring microbial protein spaces (millions or more protein sequences), models with enough flexibility can learn the inherent evolutionary patterns that natural protein sequences harbor and thus generate artificial proteins that are functionally viable like natural proteins.

To this end, ProtGPT2 was based on the GPT-2 architecture and trained on 50 million sequences spanning the entire protein space. Proteins generated by the model in return displayed propensities of amino acid sequences akin to those of natural proteins, but can still cover under-explored protein sequence regions. ProGen and its iteration (Nijkamp et al., 2023) performed similar modeling tasks, and additionally (1) allowed the inclusion of “tags” to specify protein properties for generating proteins in a more controllable fashion, and (2) experimentally verified that model-generated *de novo* protein sequences were sufficiently distinct from natural proteins but demonstrated functional viability comparable to them. Of note, while these models were typically trained to cover the universal protein space (e.g., UniRef-50), both models highlight good coverage of microbial protein properties. ProGen specifically validated the antibacterial functional property of its generated novel proteins that were comparable to natural lysozymes.

### 3.2 Protein language models for function and structure prediction

Related to, but different from the task of generating novel protein sequences, prediction-focused protein language models are primarily concerned with predicting proteins’ biological properties (e.g., 3D structures, functions) based on their AA residue sequences. Encoder-style language model architectures such as that of BERT are of particular relevance, as these models aim to learn the best representation of each token (i.e., AA) given the broader sequence context and thus can represent entire sequences in a meaningful, efficient manner. For example, Elnaggar et al. (2022) developed several LMs for protein sequences, including two auto-regressive models (Transformer-XL, XLNet) and four auto-encoder models (BERT, Albert, Electra, T5) on data from UniRef and BFD containing up to 393 billion amino acids. Transformer Uniref90 MT from the Protein BERT project can be downloaded from the project GitHub repository (https://github.com/nadavbra/protein_bert) and protein sequences are embedded using the function in the protein Bert python package. Such representations can then be fed as the input to downstream predictive models, often also realized with neural networks (NN), for various tasks.

For predicting protein structures, with scaling language models from 8 million to 15 billion parameters, the ESM-2 model (Lin et al., 2023) effectively internalizes evolutionary patterns directly from protein sequences. The learned attention patterns provided a low-resolution protein structure, corresponding to residue-residue contact maps. This was further combined with a downstream predictive module to form the ESMFold model, which offers direct inference from sequence to protein 3D structures and achieved comparable performances as SOTA protein structure prediction models (e.g., AlphaFold2). Of relevance to the microbiome, the authors applied their model to construct an atlas of predicted structures of over 600 million metagenomic protein sequences. Another group of predictive tasks aims to mine biological functions based on protein sequences. As a representative, Ma et al. (2022) focused on predicting antimicrobial peptides (AMPs) as products of the gut microbiome. They constructed the best combination over several language models, including one of the BERT architecture, to computationally mine AMP candidates from gut metagenomic studies. With additional computational filtering and experimental validation, they demonstrated that identified candidates were effective against multi-drug-resistant bacteria and demonstrated microbial membrane disruption in mechanistic studies. Such studies represent the potential of language model-aided computational efforts toward human and environmental microbiome studies for high-throughput mining of microbial structural and functional properties.

### 3.3 DNA language models at the genomic scale

The full review of DNA/genomic language models is provided in Table 2. As discussed above, microbial genomes have drastically increased scales compared to single genes or proteins. Genomic sequences also possess much sparser biological information than proteins, containing intergenic regions with both functional and junk DNA elements. The DNA sequence vocabulary also only consists of four different types of nucleotides, less than the 20 different AAs that typically constitute protein sequences. As such, language models that operate on the genome scale require additional considerations than protein models and can be further divided into two categories. The first type, often termed in literature as DNA language models, focuses on modeling DNA sequences truly on the full genome scale of organisms, e.g., DNABERT (Zhou et al., 2023), Nucleotide Transformer [NT, Dalla-Torre et al. (2024)]. As such, they adopt techniques such as specialized tokenization, alternative attention patterns, and hierarchical modeling architecture to drastically extend model contextual lengths. An important advantage of this approach is that it allows for the representation and identification of non-coding functional elements on the DNA (e.g., promoters).

Tasked with the ambitious goal of providing a generic, “foundation” model for genomes, models such as the DNABERT and NT aim to provide meaningful, contextualized representations of genome-scale DNA sequences that can be used to predict their functional properties and molecular phenotypes. Trained on genomes spanning hundreds of organisms (including genomes from microbial species) and based on encoder-style model architectures, these models are then utilized towards tasks such as predicting genomic elements (promoter, enhancer, transcription factor, epigenetic marks) and differentiating microbial species. While studies of human genomes are still the focus, they do demonstrate transferrable of learned representations across species to metagenomes, as well as improved model performance when the combination of diverse genomes was included during model training. On more reduced scales, microbial DNA language models are focused on learning the genomic pattern of specific organisms and specific genomic elements. Karollus et al. (2024) for example trained a DNABERT-like model specifically for non-coding regions up- and down-stream of gene sequences from fungal species, and demonstrated that the learned sequence representations can capture motifs and regulatory properties of these elements, in contrast to the background and non-coding sequences. While current DNA language models are still limited in training data and model capacity (the NT at its largest scale was trained with 2.5 billion parameters on 850 species) to truly operate as the foundational representation of diverse genomes, we anticipate significant progress in the near future aided by rapid development on language model scales and computational power.

### 3.4 Genomic language models contextualize genes and gene clusters

Alternatively, another group of metagenome language models examines medium-to long-range contexts between genes, often operating on the contig scale and excluding intergenic sequences. We term these as genomic models as an intermediate approach between protein and DNA language models. These models often adopt hierarchical scaffolding across genes (genes themselves are embedded by protein language models), to provide a contextualized and richer representation of genes in their broader genomic neighborhood. Gene properties such as their differential functions across microbes and genome/community-scale organization (horizontal gene transfer, operon membership) can then be further interrogated, which is not possible in protein language models where they are modeled in isolation from each other.

In comparison to full-scale DNA language models, genomic language models such as the gLM (Hwang et al., 2024) and FGBERT (Duan et al., 2024) instead focus on contig-to genome-scale organization of microbial genes (see Table 2). gLM, for example, adopts EMS-2 protein embeddings for each gene and models their genomic dependency structures on the contig (15–30 genes in length) scale. This enables, first, the enrichment of each gene’s embedding in its broader genomic texts. Genes’ “higher-order”, genome- and community-level functional properties can be further delineated that are indistinguishable from protein-scale language modeling alone, such as differential gene functions in different biomes and microbial species, as well as their self-mobility in horizontal gene transfer events across genomes. Secondly, the organization of gene clusters in linkage with each other on the genome can also be represented, whereby subsets of model attention patterns from gLM and FGBERT both demonstrated correspondence with operon memberships. The longer-scale organization of biosynthetic gene clusters is also relevant and discussed in a dedicated section as a specialized task. As population-scale studies of the microbiome often focus on gene- or pathway-level sample profiles, such genomic language models provide practical intermediate solutions to enrich microbiome studies using recent language model advancement with microbial gene elements’ broader genomic contexts.

## 4 Language models for virome annotation and virome-host interactions

The human virome consists of eukaryotic viruses that infect eukaryotic cells and prokaryotic viruses, also known as bacteriophages, that infects and replicates within bacteria and archaea. The gut virome is a vital component of the human gut microbiome, consisting mainly of viruses that infect bacteria (bacteriophages or phages), along with other viral species that may infect eukaryotic cells. The virome plays a crucial role in maintaining gut health by influencing the bacterial population dynamics, shaping immune responses, and potentially affecting the overall metabolic environment of the gut.

Metagenomic sequencing of the gut microbiome provides a wealth of information for identifying viruses, especially bacteriophages, which are key players in viral-bacterial interactions. One important method for studying these interactions is through CRISPR spacers, which serve as a molecular record of past viral infections in bacterial genomes. CRISPR-Cas systems are a bacterial immune defense mechanism that targets invading bacteriophages (Dion et al., 2021). There has been significant interest in applying recently developed protein or DNA sequence language models in virome sequence identification and annotation, as well as in building predictive models for virus-bacterium interactions based on sequence data.

### 4.1 Virome sequence annotation and identification

Annotation of viral genomes in metagenomic samples is a crucial first step in understanding viral diversity and function. Current annotation approaches primarily rely on sequence homology methods, such as profile Hidden Markov Model (pHMM)-based approaches. However, these methods are limited by the scarcity of characterized viral proteins and the significant divergence among viral sequences. To address these challenges, Flamholz et al. (2024) applied curated virome protein family (VPF) databases alongside recently developed protein language models (PLMs). They demonstrated that PLM-based representations of viral protein sequences can capture functional homology beyond the reach of traditional sequence homology methods. Their reference annotations were derived from the Prokaryotic Virus Remote Homologous Groups (PHROGs) database, a curated library of VPFs designed to detect remote sequence homology. PHROGs are manually annotated into high-level functional categories and contains 868,340 protein sequences clustered into 38,880 families, of which 5,088 are assigned to 9 functional classes. Using these data, Flamholz et al. (2024) showed that PLM-based representations of viral proteins can effectively predict their functions, even in the absence of close sequence homologs.

Peng et al. (2024) developed a viral language model (ViraLM) that adapts the genome foundation model DNABERT-2 (Zhou et al., 2023) for virus detection by fine-tuning the model for a binary classification of novel viral contigs in metagenomic data. DNABERT-2 is pre-trained on a vast array of organisms, acquiring valuable representations of DNA sequences, which is particularly useful for distinguishing viral sequences from those of other species. To adapt the genome foundation model for virus detection, they fine-tuned this model for a binary classification task with two labels: viral sequences vs. others, where they constructed a substantial viral dataset comprising 49,929 high-quality viral genomes downloaded from the NCBI RefSeq, spanning diverse taxonomic groups as positive samples. The negative data (245,734 non-viral sequences) are complete assemblies of bacteria, archaea, fungi, and protozoa, also downloaded from the NCBI RefSeq. The genomes are randomly cut into short contigs ranging from 300 to 2000 bp to minic variable-length contigs in the metagenomic data. They observed that the model initialized using the pre-trained foundation model converges faster and performs better in virus contig identification.

### 4.2 Deep learning and LLM methods for virome-host interaction

One important problem in virome research is to predict which viruses can infect which hosts, a crucial step for understanding how viruses interact with hosts and cause diseases. Virome-host interactions also play a crucial role in understanding and defining phage therapy, which uses bacteriophages to treat bacterial infections.

Currently, there are no high-throughput experimental methods that can definitively assign a host to the uncultivated viruses. A number of computational approaches have been developed to predict unknown virus-host associations. The coevolution of a virus and its host left signals in their genomes, which have been exploited for computational prediction of virus-host associations. The alignment-based approaches search for homology such as prophage (Roux et al., 2015) or CRISPR-cas spacers (Staals and Brouns, 2013; Horvath and Barrangou, 2010). Algorithms like the Basic Local Alignment Search Tool (BLAST) are commonly used to align viral sequences with host genome sequences to detect homology. This can reveal conserved regions in viral and host proteins, such as receptor-binding domains that allow viruses to enter host cells. In contrast, alignment-free methods use features such as k-mer composition, codon usage, or GC content to measure the similarity between viral and host sequences or to other viruses with a known host. By identifying which viral genomes contain sequences matching a bacterium’s CRISPR spacers, researchers can infer potential virus-host interactions. However, this approach is limited by the set of known CRISPR spacers.

As a comparison, predicting virus-host interactions based on k-mer matching and codon usage analysis is another powerful approach for identifying novel viral-bacterial interactions. Codon usage refers to the frequency with which different codons are used to encode amino acids in a genome. When a virus’s codon usage matches that of its host, it suggests that the virus has evolved to efficiently exploit the host’s translational machinery, enhancing its ability to replicate within that host. This provides critical information in predicting potential virus-host interactions. By performing joint analysis of codon usage and other genomic features, researchers can achieve more accurate predictions regarding which host species are susceptible to particular viruses.

Since these genomic features are embedded in the viral or bacterial genomes, it is possible to learn these features automatically using machine learning and AI methods. Liu D. et al. (2023) developed evoMIL for predicting virus-host association at the species level from viral sequence only. They used datasets that were collected from the Virus-Host database VHDB, (https://www.genome.jp/virushostdb/), which contains a manually curated set of known species-level virus-host associations collated from a variety of sources, including public databases such as RefSeq, GenBank, UniProt, and ViralZone and evidence from the literature surveys (Liu D. et al., 2023). For each known interaction, this database provides NCBI taxonomic ID for the virus and host and the Refseq IDs for the virus genomes. The final data set includes 17,733 associations between 12,650 viruses and 3,740 hosts that were used to construct binary datasets for both prokaryotic and eukaryotic hosts. For each of the hosts, an evoMIL model is built to predict the possible interacting viruses.

Liu D. et al. (2023) then applied the pre-trained ESM-1b model to transform protein sequences into fixed-length embedding vectors, which serve as features for downstream binary and multi-class classification. Additionally, they applied multiple instance learning (MIL) (Maron and Lozano-Pérez, 1997), where multiple instances are grouped together with a single label, and are classified as a whole. They employed attention-based MIL (Ilse et al., 2018) for each host. Specifically, for each host, they collected the same number of positive and negative viruses, and then obtained embeddings of protein sequences from viruses obtained by the pre-trained transformer model ESM-1b. To handle the input length of the PLMs, they split the protein sequences of viruses to sub-sequences for generating embeddings. An attention-based MIL was applied to train the model for each host dataset using the protein feature matrices of viruses. The resulting models can be used to predict whether a new virus interacts with a host for which a corresponding predictive model has been developed.

In addition to species-level virus-bacterium interaction prediction, Gaborieau et al. (2023) introduced a novel dataset and prediction model that focuses on phage-bacteria interactions at the strain level, utilizing genomic data of 403 natural, phylogenetically diverse, *Escherichia* strains and 96 bacteriophages. Their findings highlight that bacterial surface structures, such as lipopolysaccharides (LPS) and capsules, play a critical role in determining these interactions. Specifically, they identified bacterial surface polysaccharides as key adsorption factors that significantly enhance the accuracy of interaction predictions. This offers a valuable dataset for developing phage cocktails to combat emerging bacterial pathogens.

## 5 Deep learning and language models for prediction of biosynthetic gene clusters

Microbial secondary metabolites are chemical compounds that exhibit a broad range of functions and have great potential in pharmaceutical applications, such as antimicrobial agents and anticancer therapies. These bioactive small molecules are usually encoded by clusters of genes along the bacterial genome known as Biosynthetic Gene Clusters (BGCs). Although accurate, experimental validation of BGCs is laborious and costly. High-througput sequencing techniques, alongside advanced genome assembly algorithms, have enabled people to access the vast amount of bacterial genomic data. The genomic sequence data serves as a rich resource for BGCs mining, allowing researchers to better understand the functional potential of bacteria and discover new secondary metabolites or natural products.

Machine learning-based algorithms have been developed for the detection of BGCs in microbial genomes. antiSMASH (Medema et al., 2011) identifies candidate BGCs through multiple sequence alignment based on the profile hidden Markov model (pHMM) library constructed from experimentally characterized signature protein or protein domains, subsequently filtering these candidates using curated rules based on expert knowledge. PRISM (Skinnider et al., 2017) employs a similar approach by searching through an HMM library. ClusterFinder (Cimermancic et al., 2014) utilizes a hidden Markov-based probabilistic algorithm to identify known and unknown BGCs. Extending beyond these methods, MetaBGC (Sugimoto et al., 2019) integrates segmented pHMM with clustering strategies, making it possible to detect BGCs directly from metagenomic reads.

Despite the success of existing machine learning-based algorithms, traditional machine learning models cannot handle the long-range dependencies between genome sequences and cannot transfer knowledge from other datasets, thereby resulting in a lower power of detecting the new BGCs. Several machine learning frameworks, including those with transformer-type language modeling architecture, have been developed specifically for predicting bacterial BGCs. These models leverage advanced computational techniques to analyze genomic data and identify regions that encode for biosynthetic pathways. Many existing methods use sequences of the protein family domains (Pfams) to characterize the BGCs and bacterial genomics. Proteins are generally composed of one or more functional regions, commonly termed domains. Different combinations of domains give rise to the diverse range of proteins found in nature. The identification of domains that occur within proteins can therefore provide insights into their function.

### 5.1 Deep learning methods for BGC prediction

DeepBGC is a deep learning-based tool that uses a combination of convolutional neural networks (CNNs) and recurrent neural networks (RNNs) to predict and classify BGCs in bacterial genomes. It processes raw genomic sequences to identify BGCs and provides detailed annotations of their functional components (Hannigan et al., 2019). e-DeepBGC further extended DeepBGC to incorporate functional description of protein family domains and to utilize the Pfam similarity database in data augmentation (Liu et al., 2022). Pfam also generates higher-level groupings of related entries, known as clans. A clan is a collection of Pfam entries which are related by similarity of sequence, structure or profile-HMM. Rios-Martinez et al. (2023) developed a deep learning model that leverages self-supervised learning to detect and classify BGCs in microbial genomes. This approach aims to improve the accuracy and efficiency of BGC identification and predict the types of natural products they produce.

### 5.2 BGC prediction based on language models

Lai et al. (2023) introduced BGC-Prophet, a neural network model that leverages natural language processing (NLP) techniques to analyze genomic sequences as linguistic data, identifying patterns indicative of biosynthetic gene clusters (BGCs). This innovative approach enables the model to grasp the complex syntax and semantics inherent in genetic sequences. The input to BGC-Prophet consists of embeddings represented by 320-dimensional vectors, generated through ESM-2 (Lin et al., 2023). The model architecture integrates convolutional neural networks (CNNs) with transformer-based models, a hybrid design that effectively manages the sequential nature of DNA data, thereby enhancing the accuracy of BGC detection and classification. Table 3 compares these methods, highlighting the deep learning models and primary data sources used for training.

**TABLE 3:** Deep learning methods for BGC prediction.

Algorithm	Model	Pretraining	Level	Primary source data
DeepBGC	BiLSTM	No	Pfam	BGCs from Cimermancic et al. (2014) + MIBiG
e-DeepBGC	BiLSTM	No	Pfam	MIBiG Medema et al. (2015)
BiGCARP	ByteNet	Yes	Pfam	antiSMASH Blin et al. (2019) + MIBiG
BGC-Prophet	Transformer	Yes	Amino acids	GTDB Parks et al. (2022) + MIBiG

**FIGURE 2:**
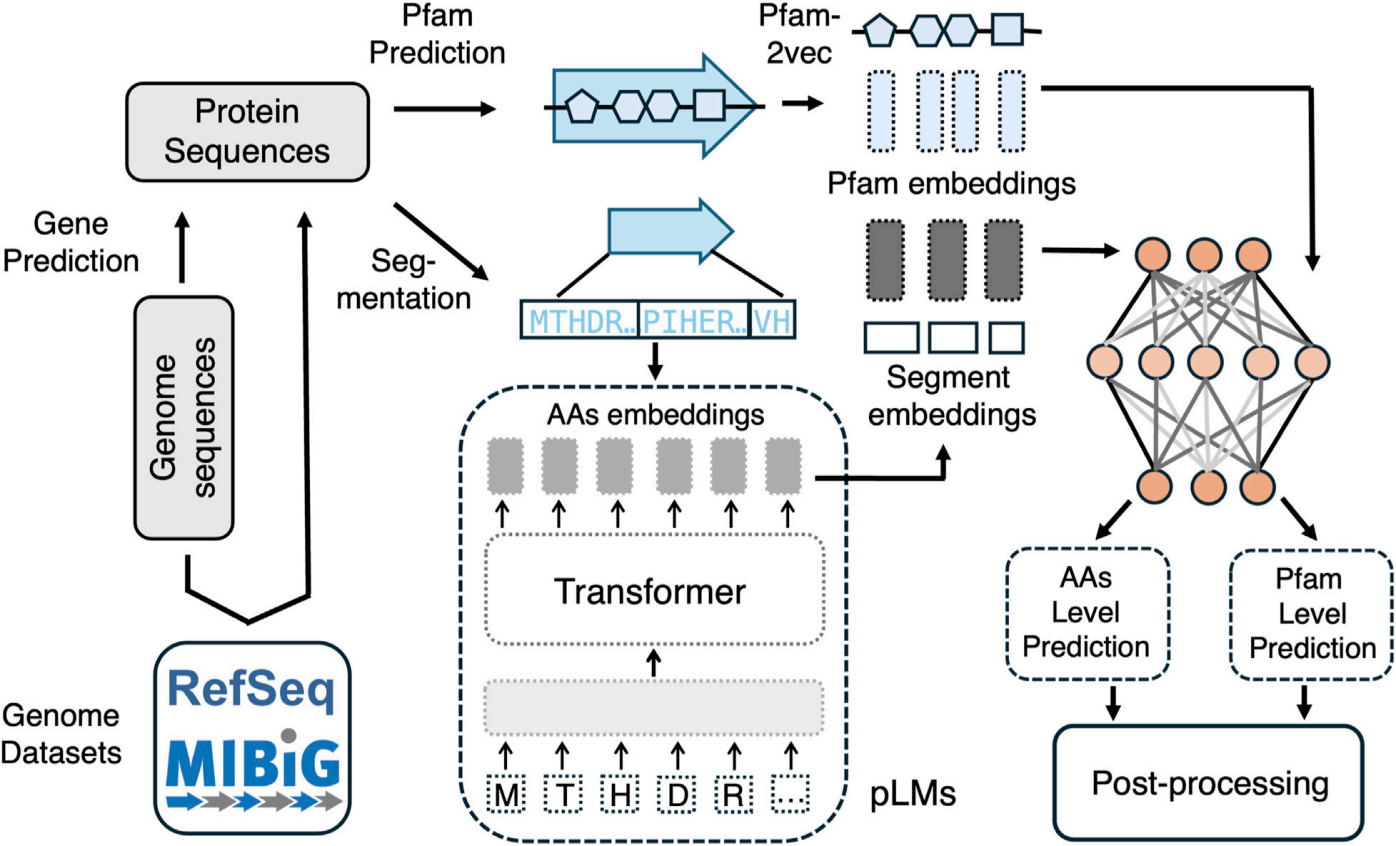
A comparison of BGC prediction based on pfam2vec embedding for Pfam level prediction and embedding based on PLMs for amino acid level prediction.

Figure 2 illustrates the differences in BGC prediction when performed at the Pfam level *versus* the amino acid level. Positive samples can be derived from segmenting amino acid sequences within biosynthetic gene clusters (BGCs) in the MIBiG database, while negative samples can be generated by randomly segmenting bacterial genomes, excluding sequences similar to known BGCs. Protein sequences can be obtained directly from genome datasets or annotated from genome sequences. For Pfam-level prediction, Pfams are first identified along the protein sequences using bioinformatics tools, and embeddings for each Pfam are generated using Pfam2vec. For amino acid-level prediction, pre-trained protein language models such as ProtBert-BFD embeddings (Elnaggar et al., 2022) are employed to embed the segmented amino acid sequences. Once these embeddings are obtained, deep learning models are applied to assign scores, indicating the probability that each embedding corresponds to a BGC.

## 6 Public knowledge integration in microbiome studies with LLMs

Due largely to the rapid development and growth of metagenomics research in the last 2 decades, it is well established that the human microbiome is associated with overall human-host health. Many of the findings that link the gut microbiome to complex diseases, such as IBD and Crohn’s Disease, can be found within individual scientific publications. Manual aggregation of these results, available in the public domain, into an organized and searchable repository would be time-prohibitive and limited to only a small subject of microbes and diseases (Badal et al., 2019). Such knowledge bases can be used for downstream analysis and discovery. NLP and text mining approaches can be used to automate this process.

Automated extraction of microbiome-disease associations from scientific text requires three steps. First is to identify the disease and microbe(s) mentioned in the text. This is known as entity extraction, where the entity is either the disease or microbe. Well-established algorithms such as Named entity Recognizers (NERs) and linguistic taggers can be used for this process. The second step is relationship extraction which aims to establish the existence of a relationship between a pair of entities (i.e., microbe-disease pair). The final step is to refine the categorization of identified relationships into positive or negative associations. Several statistical models have been developed for relationship extraction. While each step requires the use of NLP algorithms, the integration of deep learning and LMMs into steps two and three are of particular interest recently.

An early example of using deep learning in relationship extraction comes from Wu et al. (2021). In this work, the authors apply a pretrained BERE model to identify microbe-disease associations. BERE is a deep learning model initially developed for extracting drug-related associations (Hong et al., 2020). The model is pretrained using a biomedical corpus. The model converts the text into vector representation using word embeddings with sentences represented as 200-dimensional concatenations. Then the recurrent neural network encodes short- and long-range dependencies, as well as semantic features using gated recurrent units (GRUs). Finally, a classifier performs prediction. The prediction task has four possible labels: positive in which the microbe’s presence will increase when disease occurs, negative in which the microbe’s presence will decrease when the disease occurs, relate when the microbe-disease pair occurs together but the relationship cannot be determined, and NA when there is no relationship description in the text. The model requires a large amount of training data. Although, the gold standard of manual curation is difficult and costly. The authors implement a transfer learning silver standard corpus, learned with automated tools but potentially with error, first and then fine tune with the gold standard manually curated corpus. This transfer learning approach results in a reduction in the error rate.

Deep learning models like the one just described have been recently refined to use LLMs like GPT-3 and BERT (Karkera et al., 2023). The principal advantage of using LLMs in this setting is that they reduce the requirement for large amounts of training data, given that they are already pretrained with large amounts of text. The setting where no fine-tuning or training data is used is known as zero-shot learning. Karkera et al. (2023) uses the same positive, negative, relate, and NA labels as Wu et al. (2021) with their LLMs and find that zero- and few-shot learners do not perform very well, particularly with the NA label. Thus indicating that out-of-the-box implementation of LLMs for identifying microbe-disease associations is limited. The performance of generative (e.g., GPT-3) and discriminative (e.g., BERT) models improve with fine-tuning. The amount of improvement is strongly dependent on the quality of training data.

## 7 Discussion

The recent development of deep learning methods, and large language models in particular, has led to many novel applications that address significant challenges in microbiome and metagenomic research. In this paper, we have reviewed the latest applications of these methods in microbial function analysis, including the identification of biosynthetic gene clusters in bacterial genomes, annotation of virome genomes, and prediction of virus-bacteria interactions. We have also explored the use of generic LLMs, such as ChatGPT, for extracting microbe-disease associations from public knowledge. We discuss challenges and future directions below.

### 7.1 Data underrepresentation, scarcity, and quality issues

There still remain significant portions of microbial taxa, functional elements, ecologies, and environments that are poorly characterized, annotated, or cultured. These will necessarily lack representations in the “training data” databases for AI-based model. To further advance this promising research area, it is essential to focus on both the collection and annotation of datasets from multiple sources. The integration of diverse datasets—ranging from genomic sequences to environmental metadata—will provide a more comprehensive understanding of microbial communities and their interactions. However, this requires meticulous data curation, standardization, and the creation of large, well-annotated datasets that can serve as benchmarks for training and evaluating deep learning models. Specifically, for each research area as covered in this review:

- DNA, protein, and genomic language models. Models will naturally prioritize microbes and microbial genetic elements from well-studied environments and conditions (e.g., the human gut). On the other hand, current research has also demonstrated microbiome genome language models are capable of traversing genomic and protein spaces that are entirely unexplored by existing microbiome research. For example, ProtGPT2 shows that it produces not only challenging targets but also previously unreported topologies (Ferruz et al., 2022). As such, microbiome language models hold the promise to at least partially cover under-characterized microbes and genes.
- BGC identification. While the models have demonstrated significant potential, this area still faces notable challenges from data limitation. The largest experimentally validated BGCs database, MiBIG 3.0, contains approximately 2,500 entries, which is relatively small for training the AI models. To address this issue, Rios-Martinez et al. (2023) expanded the dataset by using the predicted BGCs from antismash. However, the model’s performance may be affected by the accuracy of the predicting algorithm and is subject to prediction bias. Moreover, most validated BGCs belong to Polyketide and Nonribosomal peptide classes, leaving the rest of BGC classes underrepresented. The imbalance in the training set may lead to less prediction power for less-characterized BGC types. Lastly, there is no universally accepted approach for constructing negative samples (non-BGC sequences) for training. Ideally, the negative samples should resemble true BGCs while avoiding false positives. The arbitrarily constructed negative samples may also affect the model performance. Addressing these issues of data limitation is crucial for advancing AI-driven BGC discovery and ensuring more accurate and robust predictions across diverse BGC classes.
- Virome. Unlike bacterial or eukaryotic genomes, viral genome annotations are limited by the lack of comprehensive and high-quality reference databases. This hampers the ability of language models to learn meaningful representations for virome data. In addition, viruses evolve rapidly, leading to highly divergent sequences even within closely related taxa. This makes it challenging for language models to effectively model and predict conserved functional elements or interactions. The NIH Human Virome program is expected to generate a large set of virome sequences to characterize the human virome in longitudinal, diverse cohorts across the lifespan, which can be used to develop virome-specific models. It is also possible to leverage protein structure predictions (e.g., AlphaFold) alongside sequence-based language models to improve virome functional annotations and virome-bacteria interaction predictions.
- Public knowledge integration. The automated extraction is heavily influenced by the quality of training data. Both Wu et al. (2021) and Karkera et al. (2023) note that predictive accuracy of such language models is tied to the quality of training data. Therefore, the existence of high quality gold standard corpus for training and fine-tuning are key to model performance.

### 7.2 Evaluation, interpretation, and validation of findings

Downstream interpretation and validation of findings are vital to translate the progress made with microbiome AI research into biological and clinical progress. This can be facilitated by, first, benchmarking AI models against existing data resources. For example, publicly available, high-quality microbiome cohorts, such as those constructed under the Human Microbiome Project (Integrative, 2014) and the American Gut Project (McDonald et al., 2018), should serve as real-world “silver standards” to compare recent AI models and gauge their capabilities to generate novel insights in a meaningful application setting. There have also been preliminary efforts in assembling and curating computational benchmarking tasks, such as those from Zhou et al. (2023) and Marin et al. (2023). These resources aim to compile realistic biological tasks for analysis of genomes and metagenomes with known ground truth, thus facilitating fair and meaningful comparison between AI models. However, given the nascent nature of the field, such efforts mostly focus on tasks related to the human genome. In the future, we anticipate the development of similar benchmarking resources specialized for metagenomics language models.

Second, wet-lab-based validation of new AI model discoveries is necessary, which can be realized through biochemistry-based or model-system-based evaluation approaches. For example, ProGen tested 100 AI-generated novel gene sequences (sufficiently deviating from known protein space) with cell-free synthesis and validated their bioactivity via substrate binding and fluorescence responses (Madani et al., 2023). MetaBGC purified and solved the structures of five new type II polyketide molecules as the products of characterized BGCs, two of which exhibited strong antibacterial activities (Sugimoto et al., 2019). In the future, we anticipate increasing cross-disciplinary collaborations to facilitate such practices, and in particular, the development of standardized protocols for validating AI-generated findings in real biological systems.

### 7.3 Other future directions

#### 7.3.1 Multi-domain integration

The natural large language model research has made striking progress towards multi-domain data integration, spanning data modalities such as texts, images, video, and audio (Wang et al., 2024). For metagenomic AI research, we anticipate the integration across data domains will become a similar crucial area for future research. This involves both integrating across multi-omics data modalities (e.g., metatranscriptomics, proteomics, metabolomics, host genetics) with metagenomics data, and the integration of complex biological nuances of microbiome data based on existing knowledge (interaction of microbes with host genetics, the environment, and among themselves). Successful LLM techniques such as knowledge graph integration (Pan et al., 2024) and retrieval-augmented generation (Zhao et al., 2024) can be potentially transferred for integration tasks in metagenomics AI models. Regardless, the capability of large-scale AI models promise their potential to integrate across diverse microbiome data types and existing knowledge, providing a more holistic understanding of microbial functions and interactions.

#### 7.3.2 Computation and model development

Given the model scales and the size of their training data, computations related to the development of new genomic language models can become prohibitive in order to achieve desirable model accuracy. As an example, ESM2 reported that their largest sized model (15 billion parameters) took 60 days to train over 512 NVIDIA V100 GPUs (Lin et al., 2023). We anticipate future research will develop efficient techniques to improve computational performance, especially for adapting pre-trained models towards domain-specific tasks (i.e., fine-tuning). To this end, recently efficient parameter update techniques such as adapter tuning (Houlsby et al., 2019) and Low-Rank Adaption [LoRA, Hu et al. (2021)] hold promises. For example, DNABERT-2 adopted LoRA to efficiently update the model parameters during its fine-tuning stage (Zhou et al., 2023). On the architectural front, there is a need to design models that can handle the unique challenges posed by microbiome and metagenomic data, such as high dimensionality, sparsity, and complex relationships between microbial species. Innovations in model architectures, such as graph neural networks, attention mechanisms, and hierarchical models, could play a crucial role in capturing the intricate dependencies within the data. Moreover, these models should be adaptable to the evolving nature of the datasets, allowing for continuous learning and refinement as new data becomes available.
